# A scalable 3D tissue culture pipeline to enable functional therapeutic screening for pulmonary fibrosis

**DOI:** 10.1063/5.0054967

**Published:** 2021-11-16

**Authors:** Katherine A. Cummins, Peter B. Bitterman, Daniel J. Tschumperlin, David K. Wood

**Affiliations:** 1 Department of Biomedical Engineering, University of Minnesota-Twin Cities, Minneapolis, Minnesota 55455, USA; 2 Department of Medicine, University of Minnesota-Twin Cities, Minneapolis, Minnesota 55455, USA; 3 Department of Physiology and Biomedical Engineering, Mayo Clinic, Rochester, Minnesota 55902, USA

## Abstract

Idiopathic pulmonary fibrosis (IPF) is a lethal lung disease targeting the alveolar gas exchange apparatus, leading to death by asphyxiation. IPF progresses on a tissue scale through aberrant matrix remodeling, enhanced cell contraction, and subsequent microenvironment densification. Although two pharmaceuticals modestly slow progression, IPF patient survival averages less than 5 years. A major impediment to therapeutic development is the lack of high-fidelity models that account for the fibrotic microenvironment. Our goal is to create a three-dimensional (3D) platform to enable lung fibrosis studies and recapitulate IPF tissue features. We demonstrate that normal lung fibroblasts encapsulated in collagen microspheres can be pushed toward an activated phenotype, treated with FDA-approved therapies, and their fibrotic function quantified using imaging assays (extracellular matrix deposition, contractile protein expression, and microenvironment compaction). Highlighting the system's utility, we further show that fibroblasts isolated from IPF patient lungs maintain fibrotic phenotypes and manifest reduced fibrotic function when treated with epigenetic modifiers. Our system enables enhanced screening due to improved predictability and fidelity compared to 2D systems combined with superior tractability and throughput compared to 3D systems.

## INTRODUCTION

Idiopathic pulmonary fibrosis (IPF), a lung disease with no cure, is attributed to dysregulated cell function that progresses on a tissue scale.^1,2^ Limited success in drug development coupled with therapies that slow but cannot stop fibrosis^3–5^ requires patients to undergo lung transplant or succumb to disease within an average of five years.^6,7^ Further, as chronic alveolar epithelial injury frequently precedes an IPF diagnosis, age of onset is predicted to decrease, while case numbers increase with rising global air pollution^8,9^ and the ongoing SARS-CoV-2 pandemic.^10,11^ Deficiency of effective treatment options, limited availability of lung transplants, and this projected spike in occurrence indicate a clear need for investment in more effective therapies to improve patient outcomes. Major limitations to therapy development are the insufficient disease models used in benchtop research and therapeutic screening alike. Established models have proven incapable of accurately predicting therapeutic response, partly attributable to an inability to reflect biological aspects of the disease. Consequently, IPF clinical trials have a 1% success rate^12^ compared to an average 10% rate for all small biologics^13^ and there is a foremost need to improve these models. A multitude of biologic and technologic advancements will be critical in this pursuit of models with improved pharmaceutical predictability.

One biological feature not readily captured by traditional IPF models is the loss of homeostatic control over wound healing, a result of pathologic feedback loops.^14–17^ The gold standard model, the bleomycin-injured mouse, has repeatedly proven to be poorly predictive of pharmaceutical success as it produces resolving, not progressive, fibrosis.^12,18,19^ While bleomycin-injured mice do not replicate the irreversible, worsening progression in human patients, this model captures a second critical biologic aspect: studies demonstrate a robust ability to quantify scarring in a manner difficult to replicate *in vitro.* Measuring tissue-level remodeling is imperative considering microenvironment dysregulation defines the disease. Ideally, an IPF model would facilitate quantification of matrix remodeling mechanisms, including extracellular matrix (ECM) deposition,^1,20^ microenvironment cross-linking,^15^ degradation of various structural proteins,^21,22^ and wound contraction.^15,23^ Each dysregulated mechanism contributes to disruption of respiration, by preventing lung expansion and gas exchange, and are therefore important to replicate.

Unfortunately, these phenomena are poorly represented in high-throughput platforms utilized in drug screening, including both 2D culture systems and three-dimensional (3D) organoids. Culture performed on traditional glass or polystyrene limit studying tissue functions while also providing a physiologically irrelevant environment. Instead, fibroblasts experience an overly stiff environment devoid of 3D or ECM signals and subsequently produce large quantities of matrix proteins regardless of pathology or phenotype.^24^ Alternatively, co-culture spheroids produced through a suspension of epithelial cells and fibroblasts demonstrate relevant tissue patterning and enable studies of relevant co-culture effects while limiting 2D interactions but are limited in their ability to recapitulate stromal cell, tissue-level functions in fibrosis.^25,26^ To overcome these missing dysregulated mechanisms, basic research studies of fibroblast function have been performed for decades in collagen hydrogels. These macroscopic contraction assays^27,28^ have been used to determine the pathways involved in and the effect of inhibitors on pulmonary fibrosis.^29–31^ The platforms experimental power, however, is restricted by large cell volumes and material required for casting these macrogels. Reduced throughput of hydrogel culture has correspondingly limited utility for screening applications. The demand for 3D models is complicated by the need to retain the high-throughput culture for critical drug screening applications and to mimic the throughput enabled by traditional 2D screening and automated pipetting systems. As demonstrated, these factors are frequently at odds and throughput is often sacrificed when moving to higher dimensions.

**FIG. 1.**
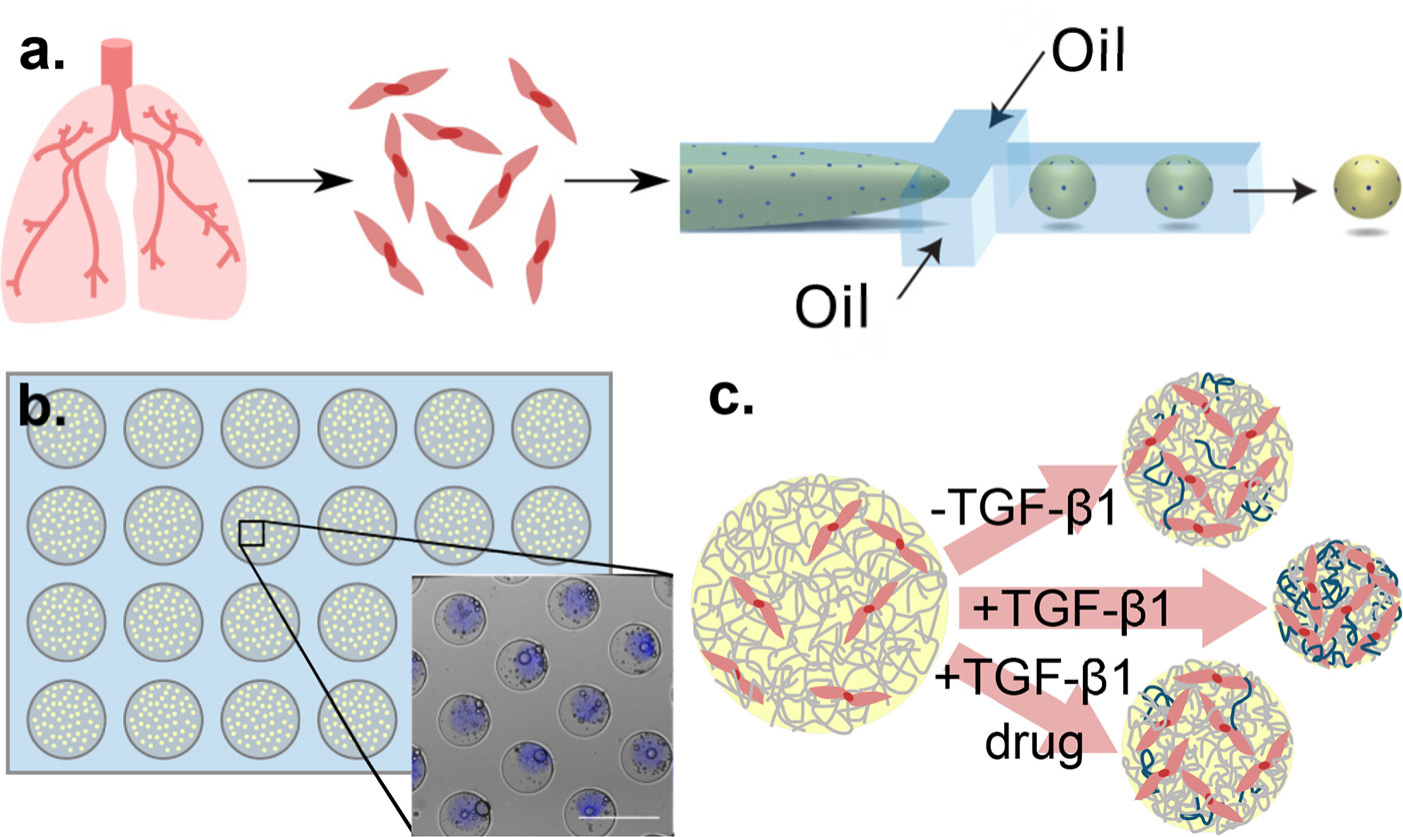
Miniaturization of the traditional large-scale hydrogel contraction assay enables high-throughput therapeutic screens as well as personalized medicine screens on a functional level. (a) Fibroblasts isolated from human lung tissue are encapsulated in collagen droplets using a flow-focused microfluidic device and off-chip fibrillization. (b) Monodisperse collagen microtissues (denoted by encapsulated blue beads) settle into a 24-well plate containing over 300 agarose microwells, enabling high-throughput culture of discrete microtissues. Scale bar 500 *μ*m. (c) Microtissues containing encapsulated normal fibroblasts can be treated with transforming growth factor-β1 (TGF-β1) to produce a fibrotic phenotype that enhances compaction and matrix deposition. These activated cells can be additionally treated with therapeutic candidates to assess efficacy in attenuating the fibrotic phenotype.

To meet this technical challenge, we developed a novel culture system that captures critical features of the IPF microenvironment, including 3D cell–cell and cell–matrix interactions and recapitulation of IPF biological mechanisms, while enabling a high-throughput workflow. To perform molecular screens of fibroblast function, we leverage microfluidic technology to encapsulate lung fibroblasts in hydrogel droplets, producing ECM droplets with flow-focusing microfluidics and off-chip thermal fibrillization [Fig. 1(a)] coupled with a microwell culturing system [Fig. 1(b)]. We additionally developed several assays to quantify hallmark fibrotic functions, including aberrant ECM remodeling and increased stromal cell contractility, both of which result in tissue stiffening and impede gas exchange. Platform validation and utility were demonstrated through repeated findings with isolated primary patient cells and the response of transforming growth factor-beta 1 (TGF-β1)-activated microtissues to FDA-approved therapeutics [Fig. 1(c)]. Finally, owing to enhanced throughput and minimal resources required per replicate, the proposed system provided the opportunity to systematically test small populations of primary fibroblasts derived from IPF patients with vastly improved statistical power. We further demonstrated a pharmaceutical screen with patient primary cells, which enhances disease relevance, and indicates potential use in personalized medicine.

## MATERIALS AND METHODS

### Study approval

#### Cell culture

Normal human lung fibroblasts (NHLFs, Lonza) were cultured in a Dulbecco's modified Eagle's medium (DMEM) supplemented with 10% fetal bovine serum (FBS, Millipore), 2 mM L-glutamine (Gibco), and 1× antibiotic–antimycotic and used for experiment between passage 4 and 7. Cryopreserved patient derived fibroblasts, isolated as previously described,^32^ were cultivated in the same medium and utilized in experiments between passages 2 and 4. We released all cell types with 0.05% trypsin-EDTA and neutralized trypsin with respective growth media, subculturing NHLFs at a ratio of 1:5 and dividing patient isolated fibroblasts 1:3. Fibroblasts were isolated from IPF patients receiving a lung transplant or from non-cancerous tissue adjacent to tumor resections (supplementary material, Table 1). All fibroblasts were cultured in an experimental culture media with reduced serum at a final concentration of 2% FBS. To simulate IPF *in vitro* with normal cells, we activated NHLFs with a single supplement of 2 ng/mL TGF-β1 24 h after tissue fabrication.

#### Microtissue fabrication

Our laboratory group^33,34^ and others^35,36^ have previously established off-chip fibrillization methods of droplet emulsions to fabricate collagen microtissues. Briefly, we buffered high concentration rat tail collagen I (Corning) with 10× Dulbecco's phosphate-buffered saline (DPBS), neutralized the solution to pH 7.4, and diluted it to a concentration of 6 mg/mL. We then resuspended fibroblasts in the aqueous collagen to a final concentration of 1.5 million cells/mL. At 4 °C, the collagen solution was partitioned into droplets by a continuous oil phase (FC-40 with 2% 008-FluoroSurfactant, Ran Biotechnologies) using a flow-focusing polydimethylsiloxane (PDMS) (Dow Corning) microfluidic device, produced through standard soft photolithography protocols. We collected constructs in a low-retention Eppendorf tube, and once all solution was partitioned, droplets were polymerized for 20 min at room temperature before removing the oil phase and resuspending the microtissues in experimental media.

#### Microwell fabrication and microtissue culture

Microwells 300 *μ*m in diameter and depth were patterned radially in 24-well plates using our established protocol.^37^ Briefly, we pre-coated standard tissue culture plates with a thin layer of agarose and dehydrated the gel to form a film on the well bottom. We then pipetted molten 2% agarose into a well and placed plasma-treated PDMS stamp into the solution at an angle, wetting the entire surface. Once fully polymerized, we gently removed the molds from the hydrogel and sterilized the microwells with EtOH before washing and hydration with appropriate media. After microtissue fabrication, we suspended cell-laden constructs in media and manually pipetted them into wells. Half media changes took place every third day, in which media with 2× additives replaced half of the media removed from the well.

#### Immunofluorescence staining

To perform standard immunofluorescence staining, we first collected microtissues in low-retention Eppendorf tubes and fixed with 3.7% formalin overnight at 4 °C. We then removed formalin and washed samples twice with DPBS prior to quenching with 0.1 M glycine. After neutralizing any residual formalin, we blocked and permeabilized constructs with 10% FBS and 0.1% Triton X-100 for 1 h at room temperature. The tissues were then incubated with primary antibody (mouse anti-alpha smooth muscle actin) (1:200, Sigma F3777), rabbit anti-fibronectin (1:500, Abcam ab23751), mouse anti-procollagen 1α2 (1:100, Santa Cruz sc-166572), mouse anti-lysl oxidase like 1 (1:100, Santa Cruz sc-166632), and rabbit anti-lysl oxidase like 2 (1:100, Thermo 702694) at 4 °C overnight. After incubation, we washed out antibody from microtissues four times with DPBS before incubating constructs with 20 *μ*M Hoechst 33258 (Thermo) for nuclear visualization along with secondary antibodies (1:500, Jackson) and/or phalloidin (1:100, Santa Cruz) at 4 °C overnight. For the supplementary TGF-β stimulation experiment, we stained for phosphorylated Smad2 (Cell Signaling #3108). After four additional washes, we moved the constructs to a 96-well plate and a Zeiss Axio Observer was used to obtain immunofluorescence images for protein expression analysis.

#### MMP-2 ELISA

In order to quantify the matrix metalloproteinase-2 synthesized and secreted by fibroblasts encapsulated in microtissues, we collected culture media from control and TGF-β1-activated four days after tissue fabrication. We analyzed the media samples with an MMP-2 ELISA kit (Thermo) to assess the protein in solution for each condition. Concentrations were normalized to the number of cells cultured within the well, assessed by releasing cells tissue digestion with collagenase IV (Thermo), cell dispersion with 0.05% trypsin with EDTA additive, and subsequent cell counting with an automated hemocytometer.

#### Viability staining

For viability staining, we collected constructs in Eppendorf tubes and washed with them warmed DPBS after settling. Microtissues were then incubated with a staining solution of 5 *μ*M DRAQ5 (Invitrogen) and 5 *μ*M calcein AM at 37 °C for 20 min. After staining, we moved constructs to a 96-well plate and imaged with a Zeiss Axio Observer and the number of live cells compared to the total number of cells per droplet.

#### Approved and experimental therapeutics studies

Nintedanib esylate (VWR) was reconstituted in dimethyl sulfoxide (DMSO) at a stock concentration of 12.3 mM and pirfenidone (VWR) reconstituted in 2.5% DMSO at a stock of 27 mM and stored at −80 °C until use. For the FDA approved therapy studies, we activated NHLFs with TGF-β1 24 h after encapsulation and treated cells with varying concentrations of therapeutics the following day. We collected tissues on the fifth day, performing viability staining on half of constructs and fixing the remaining tissues for immunofluorescence staining. Pracinostat (Thermo) (+)-JQ1 (Millipore), SRT-1720 (VWR), SGI-1776 (Neta Scientific), and UNC-669 (Neta), SP-2509 (Neta) were reconstituted in DMSO at a 15 mM stock concentration and stored at −80 °C until use. We treated encapsulated patient cells with either 250 or 1000 nM 24 h after fabrication. On the fourth day of culture, constructs were collected for viability and immunofluorescence staining. All normalizations for drug studies were made with a DMSO carrier control. For the supplemental experiment, cells were treated with 10 *μ*M of either TGF-β1 receptor 1 inhibitors GW788388 (Selleck) and SB525334 (Selleck).

#### Image acquisition and analysis

We used a Zeiss Axio Observer for all fluorescence imaging, while analysis was performed with a custom, automated Python script. Briefly, background subtraction, brightness enhancement, and contrast adjustment were performed prior to image thresholding to create masks of each microtissue. We subsequently recorded shape descriptors, population statistics, and cell counts for each droplet. The mean fluorescence intensity is the average pixel intensity value across the microtissue. To normalize these values, background fluorescence, set as the tenth percentile intensity value of each construct, was subtracted from the mean intensity and the resulting value was averaged against cell number.

#### Statistical analysis

Outliers, determined as values three standard deviations above or below the mean, were removed from data sets. GraphPad 8 was used to perform appropriate statistical analysis on each data set. Paired Student's t tests were used to compare TGF-β1-treated constructs to non-treated control tissues for single timepoints. A nested Student's t test was used to compare function of fibroblasts from IPF patients to cells isolated from control tissues. One-way ANOVA (analysis of variance) with *post hoc* Tukey test was used to compare across multiple timepoints and for drug studies. We repeated all experiments three times and found the same trends as described. A 95% confidence interval of the mean is shown for all representative data sets except for the MMP-2 ELISA data. For significance, * represents a p-value less than 0.05, ** less than 0.01, *** less than 0.001, and **** less than 0.0001.

## RESULTS AND DISCUSSION

### Amplified ECM remodeling occurs after fibroblast activation in collagen microtissues

Scarring in IPF patient lungs persists continuously in feedback loops that extend beyond tissue restoration required for healing, ultimately leading to a loss in lung compliance and impaired gas exchange.^15,23^ Homeostatic control over matrix deposition is integral in wound healing but becomes dysregulated in fibrosis.^14,16,38^ Additional aberrant remodeling occurs through amplified microenvironment cross-linking^1^ as well as stromal cell contraction, stiffening tissue above physiologic ranges.^16,23^ Finally, scarring is accompanied by disruption of the basement membrane that supports the epithelium, further altering tissue architecture.^22^ To recapitulate fibrotic processes *in vitro* with readily available cell sources, we utilized TGF-β1 induction^29,39^ in which we stimulated normal human lung fibroblasts (NHLFs) with 2 ng/mL TGF-β1 and evaluated their remodeling capabilities. We additionally verified TGF-β1 activation through inhibition of TGF-β1 receptor 1 and subsequent staining of phosphorylated signaling molecules in the pathway (supplementary material, Fig. S1).

**FIG. 2.**
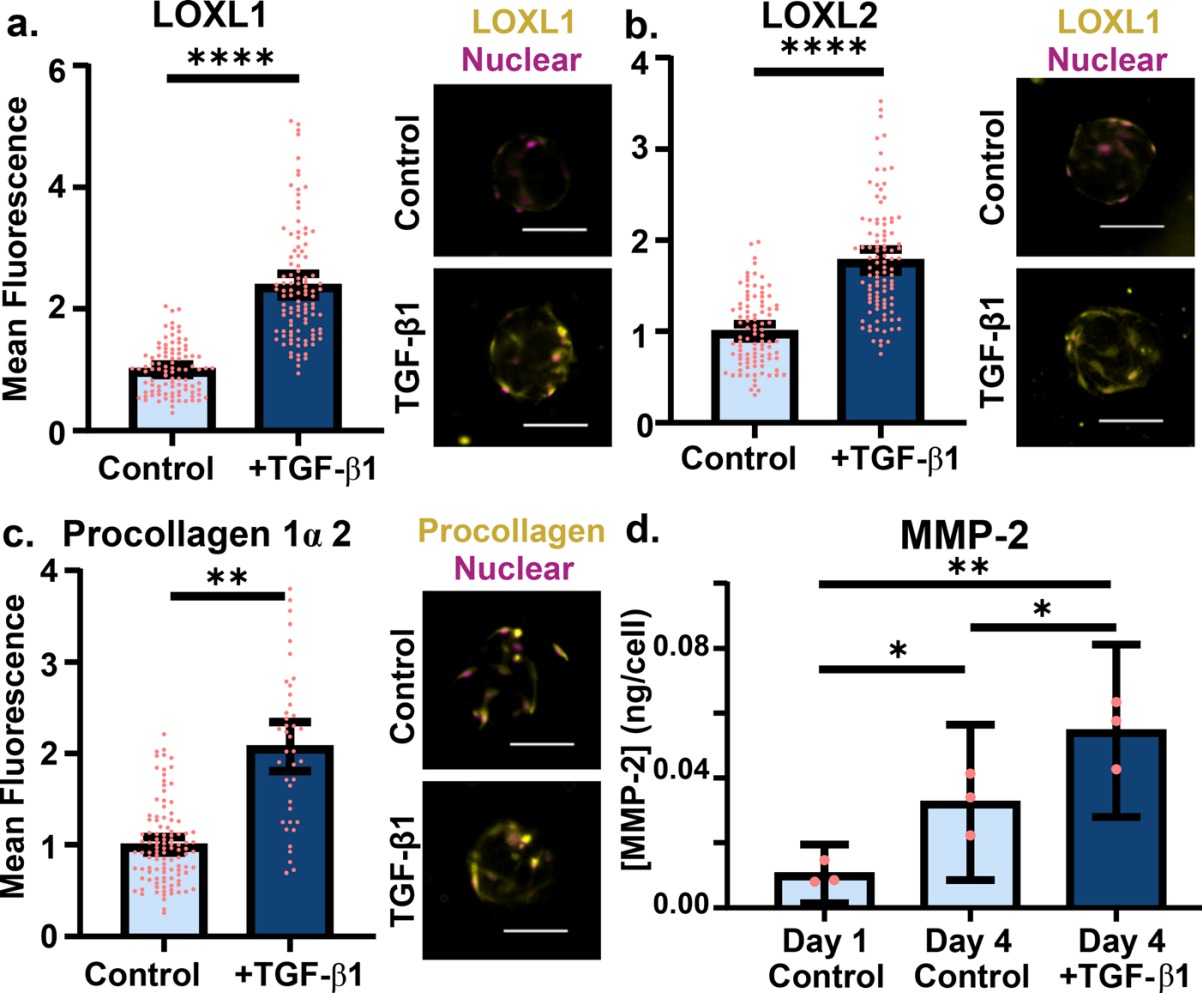
Amplified ECM remodeling occurs after fibroblast activation in collagen microtissues. (a) Lysl oxidase like protein 1 and (b) protein 2 (yellow) were stained and quantified. Mean fluorescence values were normalized by cell number [quantified by nuclear count (pink)]. (c) After 4 days of culture, fibroblasts were stained for procollagen 1α2 (yellow), a proxy indicating collagen I synthesis, and Hoechst to visualize nuclei and obtain a cell count for normalization. (d) NHLFs encapsulated in microtissues secreted MMP-2, quantified with an ELISA, and normalized by cell number. Experiment performed three times, with the average of each replicate depicted with a circle; SEM (scanning electron microscopy) shown (pink) (scale 100 *μ*m; points represent individual microtissues from single experiment).

ECM remodeling was first assessed through the visualization of ECM cross-linking proteins, a key contributor to the lung stiffening and scarring that occurs in fibrosis. Notably, lysyl oxidase like 1 (LOXL1) and 2 (LOXL2) proteins that cross-link collagen are upregulated in IPF patients^40,41^ and were found to be similarly upregulated in our activated NHLF model. After TGF-β1 stimulation and subsequent culture for 3 days, we found both LOXL1 [Fig. 2(a)] and LOXL2 [Fig. 2(b)] were expressed at significantly higher levels in TGF-β1-activated NHLFs when compared to control fibroblasts and normalized by cell number (p < 0.0001). We observed more than double the signal intensity for LOXL1 staining and greater than 1.5 times the signal when quantifying LOXL2 staining intensity.

Enhanced collagen 1 deposition^29^ is an additional hallmark of IPF and other fibrotic diseases; to visualize and assess collagen production by the cells in our collagen system, we quantified expression of procollagen 1α2, a collagen 1 precursor, as a proxy. When comparing TGF-β1-activated fibroblasts to untreated cells, we observed a strong response to stimulus when quantifying procollagen 1α2 in a twofold higher signal [Fig. 2(c)], and a statistically significant difference when quantifying mean fluorescence per cell per droplet (p < 0.01). While fibroblasts in stiff ECM-lacking environments produce collagen readily,^42^ we believed a lack in procollagen expression in the control may be a result of cells in physiologic stiffnesses experiencing a highly collagen-rich environment in the culture platform,^43^ a cue ignored in our induced fibroblasts.

Finally, while counterintuitive to elevated ECM deposition and microenvironment stiffening, aberrant remodeling also occurs through amplified matrix metalloproteinase secretion. Specifically, matrix metalloproteinase-2 (MMP-2) is a collagenase responsible for cleavage of a wide variety of ECM substrates and is found at elevated levels in lung fibrosis patient's bronchial lavage^22^ and in *in vitro* culture.^44^ When quantifying protein excreted per cell, we found that 1.67 times more MMP-2 was secreted by TGF-β1-activated fibroblasts when compared to unstimulated cells [Fig. 2(d), p < 0.05]. MMP-2 upregulation is of importance as it is largely associated with basement membrane disruption that leads to disordered tissue structure and fibrogenic proliferation.^21,44^

### Increased fibroblast contractility is observed with fibroblast activation in collagen microtissues

Beyond fibrous matrix deposition and cross-linking, lung stiffening occurs through enhanced mechanical forces applied to the microenvironment by highly contractile myofibroblasts.^15,16^ This fibroblast functionality has been investigated with the previously described large collagen hydrogels but are restricted by the limited number of experimental conditions and replicates that can be tested. These large hydrogels are additionally incompatible with standard biological staining assays due to size; both reagent diffusion and optical limitations reduce the readouts possible in standard models. Miniaturization of these large gels with the microtissue platform, however, overcomes these barriers.

**FIG. 3.**
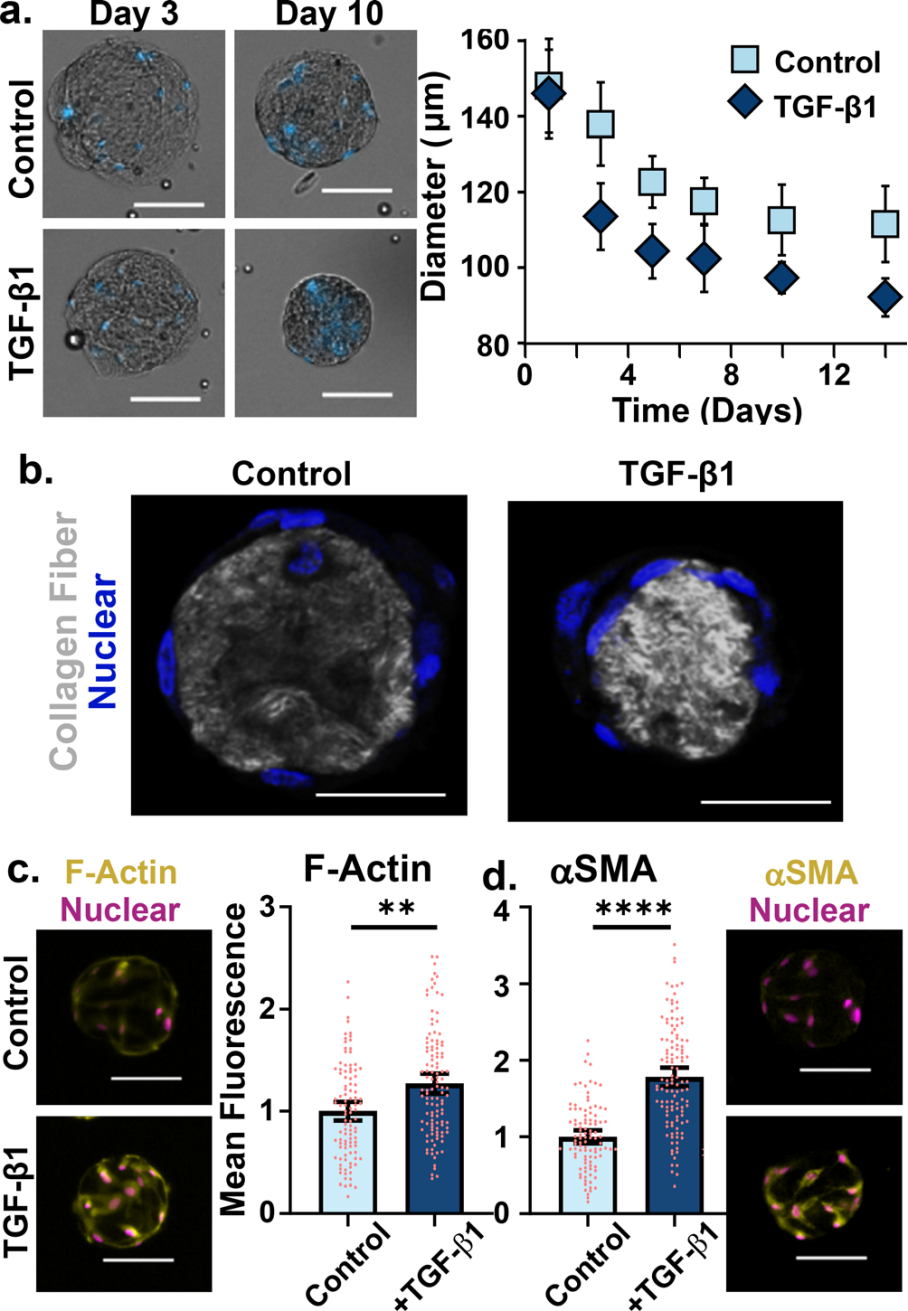
Increased fibroblast contractility is observed with fibroblast activation in collagen microtissues. (a) NHLFs were encapsulated in collagen microtissues and cultured for two weeks. During this time, cells spread and contracted, compacting collagen and reducing size of the microtissue, as quantified by a decrease in projected tissue area [nuclei stained with Hoechst (blue), >50 constructs per condition, 95% CI shown, scale bars 100 *μ*m]. (b) Compacted collagen (gray), as depicted by greater intensity of second harmonic generation signal, was visualized with a multiphoton microscope and nuclei were stained with Hoechst (blue, scales bar 50 *μ*m). (c) 1 week after TGF-β1 stimulation, NHLFs were stained to visualize filamentous actin and alpha smooth muscle actin. Average intensity per droplet was quantified and a normalized by cell number (scale bars 100 *μ*m, points represent individual microtissues from a single experiment).

When treated with TGF-β1, we observed increased compaction of collagen microtissues by activated fibroblasts, quantified by a statistically significant decrease in projected area of constructs, and sustained over two weeks [Fig. 3(a)]. This compaction likely reflects both fibroblast contractility as well as capacity to reorganize and remodel collagen fibrils. After an initial sharp deviation in tissue area, encapsulated fibroblasts begin to plateau to a steady-state construct size, when the tissues may have reached a threshold stiffness. Similar compaction profiles in which there is a large amount of compaction in the first 24 h that levels off with time are evident in the seminal findings of the large-scale hydrogel contraction assay reported by Bell *et al.*^27^ In addition, the sustained fibrotic response after a single dosing with exogenous growth factor is highly relevant for modeling the pathologic feedback loops of IPF in which fibrotic functions further induce and promote a fibrotic phenotype in cells.^16,39^ For high-throughput screening of ECM remodeling, the 1-week timepoint in which constructs had approached homeostasis may be relevant; investigations of short-term contraction may be feasible on shorter timescales, as differentiation between control and treated cells began within 48 h. Using a multiphoton microscope to capture second harmonic generation data, we visualized collagen fibers within control and TGF-β1 stimulated microtissues [Fig. 3(b)]. This revealed qualitatively brighter, denser signal indicating increased collagen fiber density, likely a result of large change in construct volume. A 37-fold reduction in volume occurred in TGF-β1-activated constructs (and possible 37-fold times increase in density) vs an 18-fold reduction in control tissue volume when compared to the starting construct with a 300 *μ*m diameter.

In addition to quantifying a difference in collagen droplet compaction as a cellular function, we assessed expression of proteins that enable this contractile force. After 1 week of culture, we found two contractile proteins were upregulated in TGF-β1-activated fibroblasts. The main marker for the fibrotic myofibroblast phenotype, alpha-smooth muscle actin (αSMA),^15,30^ was found to be expressed at significantly higher levels (p < 0.0001) after 1 week in culture, indicating a transition to a pathologic phenotype [Fig. 3(c)]. Comparatively, when quantifying filamentous actin expression, we observed fibroblasts producing significantly higher levels of F-actin after the same culture period when activated with TGF-β1 [Fig. 3(c)]. While not the hallmark contractile protein of diseased fibroblasts, F-actin is a key component of applying force from the cell (through its actin cytoskeleton) through adhesions, and into the surrounding microenvironment.^45^

### TGF-***β***1-activated microtissues respond to FDA-approved therapeutics

**FIG. 4.**
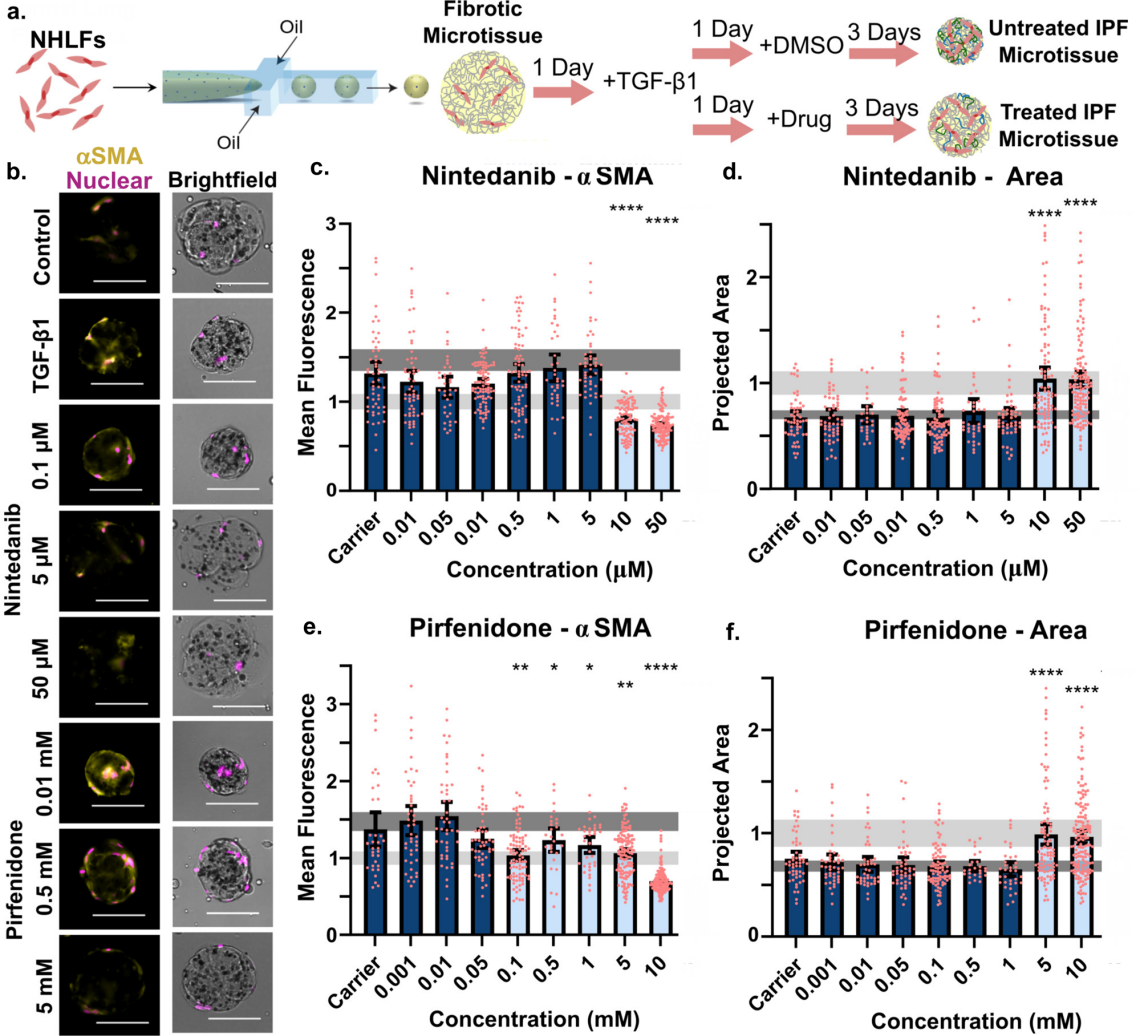
TGF-β1-activated microtissues respond to FDA approved therapeutics. (a) TGF-β1-activated NHLFs were treated with nintedanib and 3 days later collected and stained for αSMA. (b) NHLFs encapsulated within collagen microtissues were stained for αSMA (yellow) or stained with Hoechst to visualize nuclei (pink). (c) TGF-β1-activated fibroblasts treated with nintedanib and the average αSMA signal intensity and (d) projected area of the compacted microtissues were quantified for each condition. Conditions were normalized to the unactivated control, represented by the light gray bar (95% CI of the mean). Displayed statistics compare conditions to untreated, TGF-β1-activated NHLFs, represented by the dark gray bar (95% CI of the mean). (e) Activated NHLFs were treated with pirfenidone, and the average αSMA signal intensity and (f) projected area of the compacted microtissues were quantified for each condition. (ANOVA with *post hoc* Tukey analysis was performed, scale bars 100 *μ*m; points represent individual microtissues from a single experiment.) (see also supplementary material, Fig. 2).

After developing pathologically relevant metrics for characterizing fibrosis in collagen microtissues, we set out to test drug screening utility, employing pharmaceuticals used for disease management. In these experiments, we treated TGF-β1-stimulated fibroblasts with FDA-approved therapeutics nintedanib and pirfenidone across a wide concentration range. We then quantified compaction and compared treated, activated cells to unactivated fibroblasts and to non-treated activated cells to assess drug efficacy in microtissues [Fig. 4(a)]. Treatment with nintedanib, a tyrosine kinase inhibitor for adenosine triphosphate (ATP)-ATP-binding sites in receptors that stimulate myofibroblast activation (fibroblast growth factor receptor, platelet-derived growth factor receptor, and vascular endothelial growth factor receptor),^46^ ranged from 0.01 to 50 *μ*M. Pirfenidone, which reduces a variety of fibrogenic functions including collagen fiber deposition and sensitivity to TGF-β1,^4^ was used at concentrations ranging from 0.001 to 10 mM.

Prior to fibrotic function characterization, we assessed viability through calcein AM staining and found that while viability was consistent for all nintedanib concentrations, we observed stark differences in cellular phenotype at 50 *μ*M. NHLFs appeared less elongated, exhibited fewer branching points, and stained with a punctate pattern (supplementary material, Fig. S2), indicating a reduction in overall cell health despite not reaching cytotoxic levels. When assessing viability of cells treated with pirfenidone, we found cells remained highly viable (greater than 90%) in all conditions except for cells treated with the highest dose. After viability assessment, we compared the effects of each intervention on fibrotic function in TGF-β1-activated microtissues through αSMA staining and quantification of microtissue compaction through projected area measurement [Fig. 4(b)]. When treated with 10 *μ*M of nintedanib, we observed that TGF-β1-activated microtissues expressed similarly low levels of αSMA and equally limited compaction as non-activated microtissues [Figs. 4(c) and 4(d)]. This dose fell well below concentrations at which we observed reduced viability or phenotypic changes. Notably, this finding contradicts the majority of 2D studies that report a response to nintedanib at 400 nM to 1 *μ*M,^46^ whereas 10 *μ*M is a comparable concentration to those required for fibrosis reduction in other 3D models and *in vivo* systems.^47,48^ This phenomenon of 3D drug resistance is frequently observed in chemotherapy development for cancer treatment^49^ and indicates potential value in screening IPF therapies in a 3D context.

Pirfenidone-treated cells, meanwhile, demonstrated reduced αSMA expression at lower concentrations than were required to attenuate droplet compaction. When quantifying intensity of αSMA staining, we observed a significant effect at 0.1 mM of pirfenidone [Fig. 4(e)] but found 5 mM of pirfenidone was required to reduce compaction [Fig. 4(f)]. This discrepancy further indicates the importance of assessing IPF drug effects in 3D and not relying solely on 2D immunofluorescence staining. Reported values indicated cells respond to pirfenidone in 2D at concentrations ranging from 0.1 to 1 mM,^47^ comparable to the values at which we observed reduction in αSMA staining but again, well below the values at which a tissue-level effect could be observed. Finally, unlike nintedanib, we found a dose-dependent response to pirfenidone when assessing αSMA staining intensity data.

From a technology perspective, these results demonstrate potential utility of microtissues for drug screening, using validated compounds as surrogate candidates. The platform enabled multiplexing of cellular-level functions, such as viability and morphology, with our described fibrosis metrics while providing enhanced statistical power. Reduction in fibrotic readouts within non-cytotoxic concentrations could be utilized in future screening to prioritize compounds for continued study and drug development. Moreover, we demonstrated the assays were tractable with a screen of over 48 conditions and controls, performed with a small batch (250 *μ*L collagen, 3.75 × 10^5^ NHLFs) of collagen microtissues. Discrete points represent individual microtissues per experiment in which more than 25 microtissues were analyzed per condition (Fig. 4). The screen was mainly limited by manual labor available for culture and by staining reagents and would be scalable for larger operations.

### Primary patient fibroblasts validate fibrosis–microtissue phenotype metrics

**FIG. 5.**
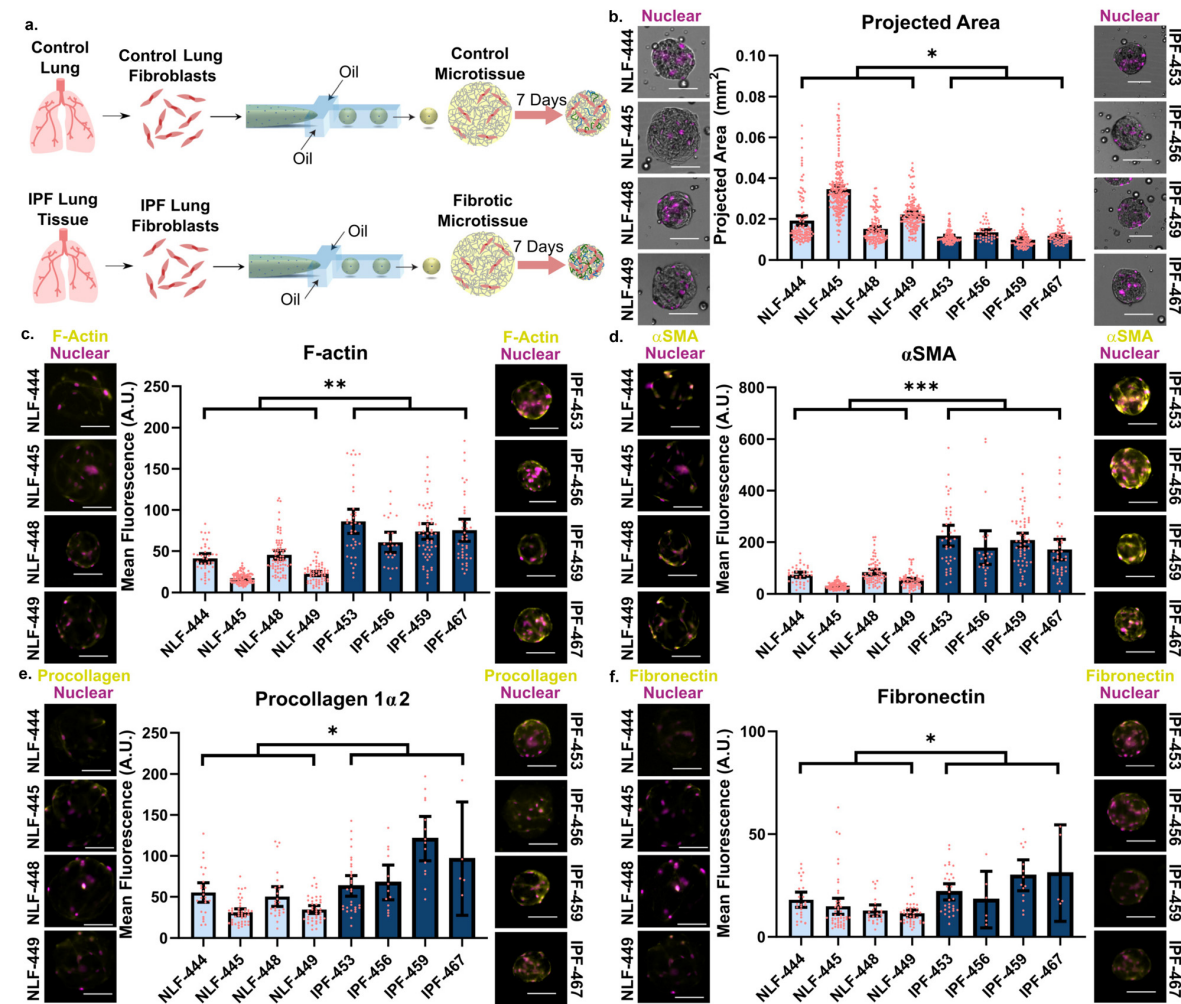
Primary patient fibroblasts validate fibrosis–microtissue phenotype metrics. (a) Fibroblasts were isolated from IPF patients receiving a lung transplant or from non-cancerous tissue adjacent to tumor resections. After expansion, cells were encapsulated in collagen microtissues at a concentration of 1.5 × 10^6^ cells/mL of collagen. (b) Microtissue compaction was quantified as projected area after 1 week. (c) Patient cells were stained for F-actin and (d) αSMA to visualize microfilament proteins. (e) Additional staining was performed to visualize ECM production and deposition of procollagen 1α2 and (f) fibronectin (95% CI shown, scale bars 100 *μ*m, points represent individual microtissues from a single experiment) (see also supplementary material, Fig. 3).

To further validate utility of the proposed system and phenotypic metrics, we encapsulated isolated fibroblasts from 4 IPF tissue samples (confirmed usual interstitial pneumonia) and from patient controls (histologically uninvolved tissue from tumor resections) (supplementary material, Table 1). We then repeated our fibrosis assays with these samples [Fig. 5(a)]. When staining for F-actin, αSMA, procollagen 1α2, and fibronectin, we found that all were constitutively expressed at higher levels in microtissues containing fibroblasts isolated from IPF patients when compared to patient controls, similar to our observations of TGF-β1-induced activation of NHLFs.

We found encapsulated IPF cells exhibited a significantly more contractile phenotype when examining cell function and protein expression. When utilizing the miniaturized compaction assay, we calculated projected areas to be significantly smaller when encapsulating IPF cells compared to control primary cells [p < 0.05, Fig. 5(a)], indicating a more contractile phenotype. In addition to tissue-level function, these fibroblasts express contractile proteins F-actin [p < 0.01, Fig. 5(c)] and αSMA [p < 0.001, Fig. 5(d)] at higher levels than lung fibroblasts from control samples. Each contractility metric was similar to the enhanced contractile phenotype observed in TGF-β1-activated microtissues. Beyond enhanced compaction, primary cells isolated from lungs of IPF patients also demonstrated aberrant microenvironment control through ECM deposition in highly fibrous environments. These IPF fibroblasts expressed an excess of procollagen 1α2 [Fig. 5(e), p < 0.05] and fibronectin [Fig. 5(f), p < 0.05] per cell when compared to control cells. Despite enhanced matrix deposition, expression of cross-linking enzymes was not found to be significantly enhanced although IPF cells appear at the upper boundary of the population distribution (supplementary material, Fig. S3).

Overall, encapsulation of patient primary cells provided robust validation of the proposed phenotypic metrics. This further confirms the relevance of the TGF-β1-activated microtissue, which exhibited largely overlapping phenotypes with microtissues formed from primary, IPF-derived cells. Use of exogenously activated microtissues, and the associated assays, could therefore enable large discovery screens for potential therapeutic leads. In IPF, there are many reports documenting the importance of the pathological ECM in driving the fibrotic phenotype.^14^ In addition, IPF fibroblasts are reported to manifest cell autonomous pathology,^32,50–53^ in accord with the differences we observed between the patient cohorts despite isolation and expansion prior to microtissue encapsulation. We hypothesized that epigenetic changes to IPF fibroblast DNA, a prevalent theory for IPF disease occurrence, progression, and maintenance,^54^ sustained the fibrotic phenotype observed in our system.

### Treatment with epigenetic modifiers inhibits intrinsic fibrotic function of IPF patient cells

Through maintenance of fibrotic functions after expansion and subsequent encapsulation of isolated primary fibroblasts (Fig. 5), it was apparent that IPF fibroblasts maintained a fibrotic phenotype independent of microenvironment cues, perhaps passed to progeny through epigenetics. We consequently proposed to reduce fibrotic functions with four top candidates from a recent 2D screen of approximately one hundred small molecule epigenetic modifiers.^55^ The first, pracinostat, inhibits histone deacetylases and, similar to reviving tumor suppressor genes, may induce anti-fibrotic transcription factors.^55^ Alternatively, (+)-JQ1 inhibits bromodomain proteins from histone binding, preventing transcriptional regulation. Among a variety of downstream effects, such inhibitors suppress a range of oncogenes, which may have roles in fibrosis.^56^ SRT-1720 activates sirtuin-1, an enzyme that aids in silencing genes governing cell regulation and stress responses.^57,58^ Finally, SGI-1776 acts as an inhibitor to proviral integration site for the murine leukemia virus (PIM) kinase. PIMs aid in cell growth and division and are involved in tumorigenesis and regulating oncogenes, and their inhibition is anti-fibrotic.^59^

**FIG. 6.**
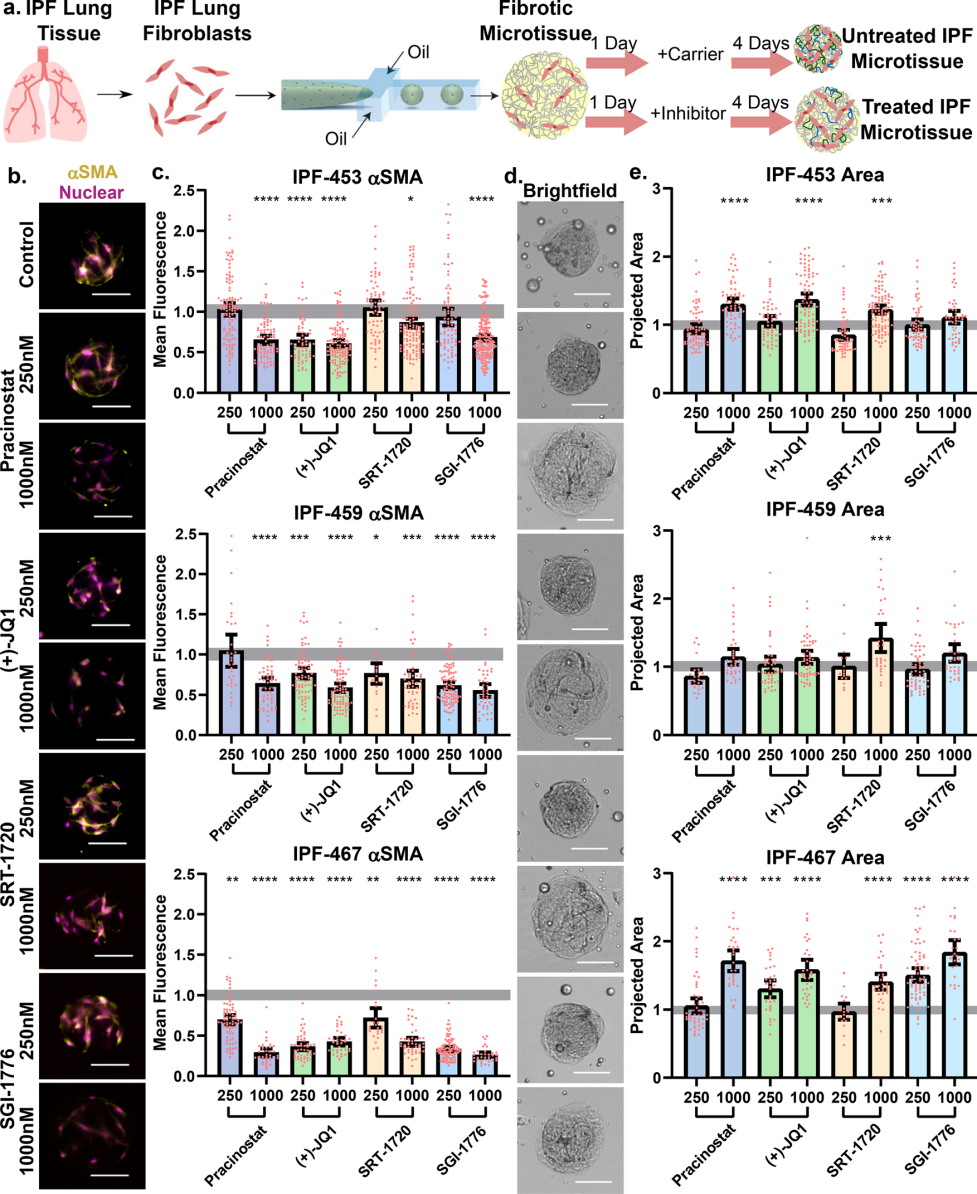
Treatment with epigenetic modifiers inhibits intrinsic fibrotic function of IPF patient cells. (a) IPF patient cells were treated 1 day after fabrication with a low (250 nM) or high (1000 nM) concentration of one of four epigenetic modifiers: pracinostat (+)-JQ1, SRT-1720, or SGI-1776. Control tissues were cultured with a DMSO carrier. Constructs were collected 4 days after treatment and fixed for remodeling quantification. (b) Representative images of fibroblasts from patient IPF-453 stained for αSMA expression (yellow). Nuclei were visualized using Hoechst (pink). (c) Fluorescence intensity of αSMA staining was quantified for each patient cell in response to each inhibitor and compared to a non-activated, carrier control microtissue tissue (gray bar). (d) Representative bright-field images of IPF-453 patient fibroblasts are shown. (e) Projected area was quantified and normalized to a carrier control (ANOVA with *post hoc* Tukey analysis was performed, gray shaded bar shows mean value with 95% CI for carrier control, scale bars 100 *μ*m, and points represent individual microtissues from a single experiment.) (see also supplementary material, Fig. 4).

Each compound was used to treat three patient lines at low (250 nM) and high (1000 nM) concentrations, and after 4 days of culture, we assessed viability and fixed constructs for fibrosis analysis [Fig. 6(a)]. Cells from IPF-456 had significantly increased doubling times once these studies were performed and were omitted from the study. After encapsulation and drug treatment, fibroblasts retained high viability (supplementary material, Fig. S4) and treated fibroblasts demonstrated a significant reduction in αSMA expression compared to untreated cells [Figs. 6(b) and 6(c)]. This robust response to each applied therapy was expected as the 2D study ranked therapies based on attenuation of αSMA signal. In addition, we observed a dose-dependent response in which higher therapy concentrations more greatly reduced αSMA than low doses.

Reminiscent of the pirfenidone-NHLF study (Fig. 4), we found that while αSMA expression was greatly reduced in many conditions, compaction was less affected [Figs. 6(d) and 6(e)]. While αSMA has long been considered a hallmark of fibrotic stromal cells, its expression has recently been found to have limited correlation with functional fibrosis hallmarks across many organs.^60^ These results coupled with our studies indicate that decreased αSMA expression may not correlate with reduced disease severity. The epigenetic inhibitor studies and the NHLF/FDA-approved therapeutics studies demonstrated utility of the microtissue compaction assay to identify therapeutic effects on fibroblast function beyond molecular signatures. This observation is unattainable in 2D studies but is also impractical in traditional, large hydrogel methodologies, indicating the microtissue system's usefulness for therapeutic screening. Beyond demonstrating technological utility, each patient sample additionally responded to a high concentration of SRT-1720 [Fig. 6(c)], perhaps indicating potential for future IPF drug development. Currently, SRT-1720 is being investigated for use in both renal and cardiac fibrosis through disruption of the TGF/Smad signaling cascade, a positive feedback loop in all organ fibrotic diseases,^57,58^ further supporting consideration for future IPF studies.

Finally, inter-patient variability was apparent for the compaction and αSMA staining assays. For example, cells isolated from IPF-467 were strong responders to each inhibitor at all doses while IPF-459 had limited response to most therapies. As IPF is by definition idiopathic and often thought of as a syndrome more than a disease of single origin, it is expected that fibrotic cells encapsulated in collagen droplets replicate patient variability in fibrotic expression (Fig. 5) while revealing patient specific drug effects (Fig. 6). In addition, we demonstrated the platform's ability to parse out these varied responses while using limited patient sources; this screen was performed with 300 000 cells per patient for each experiment and was limited mainly by personnel. Consequently, it is possible that IPF microtissues could be leveraged to screen for potential therapeutic strategies for patients who are non-responsive to traditional therapeutics produced for a broad set of IPF patient populations, although obtaining samples from non-end-stage patients may be difficult with current standards of care. Overall, however, the functional response of patient cells to the broad class of epigenetic modifiers is highly encouraging. Our results contribute to a growing body of literature hypothesizing a role for epigenetics in disease maintenance and providing additional promise for targeting various epigenetic mechanisms for novel therapeutic strategies.

## OUTLOOK

The described microtissue platform has enhanced biological relevance over 2D screening while retaining throughput that is unviable in 3D and *in vivo* models. This physiologic relevance beyond traditional culture is enabled by the soft, natural ECM microenvironment provided by the microtissues. Furthermore, microtissues are conducive to studies of remodeling via contraction that are impossible on a tissue culture plastic interface. When compared to analogous large hydrogel contraction assays, the IPF-microtissue system not only scales up experiments in terms of replicates, but also enables quantitative immunofluorescence staining that is restricted by diffusion and microscopy limitations in larger gels. Finally, in comparison with the bleomycin-mouse model, the described method has shown initial evidence of sustained fibrosis, as well as enabled therapeutic screens that reflect human patient biology. Improvements in throughput are also evident; the high number of individual microtissues that are studied per condition coupled with multiplexing compatible readouts makes the platform suitable for high-throughput screens (HTS).

Beyond the work presented here, the platform may be further fine-tuned to produce higher fidelity models capable of parsing out disease mechanisms. The system lends itself to micropatterned co-culture by coating constructs with a secondary cell type and enabling encapsulation of multiple cell types,^33,34^ facilitating studies on how heterotypic interactions and paracrine signaling impact fibrosis progression. Alternative modifications to the platform involve encapsulation of additional matrix proteins or sourcing matrix from reconstituted lyophilized patient tissue to investigate matrix effects on fibrotic function.^61^ Finally, although the described system is not the only recently developed platform for 3D IPF pharmaceutical screening,^48^ the proposed droplet platform improves throughput by reducing cell numbers and limiting construct failure due to multi-stage microfabrication or through contraction off cantilever posts. While our microtissue system improves throughput, future work could be done to optimize the proposed assays for HTS compatibility, through increasing the culture quantity and maximizing z'-factor. Throughput could be enhanced by culturing constructs with robotic pipetting mechanisms in manufactured microwell plates, as these studies were largely limited by manual handling, large well formats, and personnel. Assay z-factor, a measurement of effect size, could be maximized through further optimization of experimental time points and through rigorous testing of commercially available antibodies. Both methods would improve dynamic range of the proposed assays and aid in their translation to HTS in an industry setting.

We have demonstrated the utility of a novel 3D cell culture platform and the high-throughput, high-content metrics of IPF. IPF microtissues enable tissue-level, cell-level, and protein-level analysis of fibrotic functions when encapsulating activated normal fibroblasts or cells derived from fibrosis patients. Therapeutic studies demonstrated the utility of the platform for reducing myofibroblast function while retaining high statistical and experimental power due to throughput enacted by the system. We believe that the IPF microtissue model is exceptionally suited to aid in therapeutic development by improving the IPF drug screening pipeline. Finally, the assays and methodologies outlined here should be of significant utility to investigations of other disease spaces in which 3D culture is important and tissue remodeling is prevalent, including but not limited to other fibrotic disease, cancers that present as solid tumors, and diseases interfering with wound healing.

## SUPPLEMENTARY MATERIAL

See the supplementary material for referenced supplementary material.

## Data Availability

The data that support the findings of this study are available from the corresponding author upon reasonable request.
